# A Tale of Two Conditions: Pediatric Brugada Syndrome Unveiled—Navigating the Challenges of Coexisting Arrhythmia and Fever‐Induced ECG Pattern

**DOI:** 10.1111/anec.70009

**Published:** 2024-12-26

**Authors:** Hei‐To Leung, Sit‐Yee Kwok, Fong‐Ying Shih, Kin‐Shing Lun, Tak‐Cheung Yung, Sabrina Tsao

**Affiliations:** ^1^ Department of Paediatrics and Adolescent Medicine Hong Kong Children's Hospital Ngau Tau Kok Hong Kong; ^2^ Clinical Genetics Service Unit Hong Kong Children's Hospital Ngau Tau Kok Hong Kong; ^3^ Department of Paediatrics and Adolescent Medicine The University of Hong Kong Pok Fu Lam Hong Kong

**Keywords:** coexisting arrhythmia, focal atrial tachycardia, long QT syndrome, pediatric Brugada syndrome, subcutaneous ICD

## Abstract

**Background:**

Brugada syndrome (BrS) is an inherited channelopathy characterized by right precordial ST‐segment elevation. This study investigates the clinical and genetic characteristics of children with BrS in Hong Kong.

**Methods:**

A retrospective review was conducted at the only tertiary pediatric cardiology center in Hong Kong from 2002 to 2022, including all pediatric BrS patients under 18 years old. The diagnosis of BrS was established with a type 1 ECG pattern detected spontaneously or induced by flecainide, excluding secondary causes.

**Results:**

Eight probands of mean age 10 years old were identified. Male dominance was observed (6 boys vs. 2 girls). The mean follow‐up duration was 4.6 years (Median 3.5 years). Patients had type 1 ECG pattern either spontaneously (*n* = 4) or provoked by flecainide (*n* = 4). Fever was present in seven patients at the initial presentation, and two patients experienced aborted cardiac arrest and one had symptomatic ventricular tachycardia. All symptomatic patients received implantable cardioverter‐defibrillator placement. Five asymptomatic patients (62.5%) were diagnosed with BrS through ECG during febrile illness, and they remained asymptomatic following conservative management involving strict fever control and medication avoidance. Two patients with mixed phenotype (one with long QT syndrome and another with ectopic atrial tachycardia) required antiarrhythmics and one patient received transcatheter ablation for atrial tachycardia to achieve optimal arrhythmia control.

**Conclusion:**

Fever plays a significant role in unmasking BrS in children. Asymptomatic children with BrS managed conservatively have a favorable prognosis. Difficult arrhythmia control was found in patients with mixed phenotype.

## Graphical abstract

Fever often unmask Brugada syndrome (BrS) in children. Asymptomatic kids with fever‐induced type 1 BrS ECG generally have a good short‐term prognosis. However, managing coexisting arrhythmias in children with BrS can be challenging. Treatment should focus on the observed malignant arrhythmia, carefully monitoring side effects of anti‐arrhythmic medications.

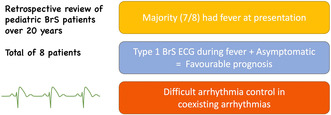

## 1 Background

Brugada syndrome (BrS) is an inherited channelopathy characterized by right precordial coved type ST‐segment elevation and potentially lethal ventricular arrhythmia. It is responsible for 4%–12% of all cases of sudden cardiac death, with a higher prevalence in young male adults (Coppola et al. 2021). However, BrS is exceedingly rare in children, as demonstrated by the limited cohort study conducted by Probst et al. (2007) which identified only 30 pediatric patients across 13 institutions in 15 years. Given its rarity, there is still a significant knowledge gap regarding the clinical presentation, risk stratification, optimal treatment approaches and prognosis specific to pediatric BrS.

## 2 Methods

This is a retrospective review involving patients over a 20‐year period (1st January 2002–31st December 2022) in the Department of Pediatric Cardiology of Queen Mary Hospital (QMH), which was subsequently translocated to Hong Kong Children Hospital (HKCH).

Our study included patients who:

1. were diagnosed with BrS, and
2. were less than 18 years of age at the time of diagnosis.

The diagnosis of BrS was established with a type 1 electrocardiogram (ECG) pattern detected spontaneously or induced by flecainide or fever, in the absence of secondary causes such as electrolytes imbalance or mechanical compression of the right ventricular outflow tract (Antzelevitch et al. 2005).

## 3 Results

Eight pediatric patients of BrS were identified over the past 20 years (2002–2022) in our single tertiary pediatric cardiac center in Hong Kong. The clinical and genetic profiles were shown in Table 1. Eight probands of median age 13.5 (3.5–15.5) years old were identified. Male dominance was observed (6 boys vs. 2 girls). The median follow‐up duration was 4.5 (2–7.5) years. Among the eight patients, one had a repaired perimembranous ventricular septal defect and atrial septal defect, while the remaining seven patients had structurally normal hearts. Patients had type 1 ECG pattern either spontaneously (*n* = 4) or provoked by flecainide (*n* = 4). Seven patients had fever at the initial presentation. Two patients had aborted cardiac arrest and one had symptomatic ventricular tachycardia at presentation. Five asymptomatic patients (62.5%) were diagnosed by ECG when they had febrile illness because of tachycardia. After fever subsided, their resting ECG revealed right bundle branch block in two patients and a type 2 BrS ECG pattern in three patients. Implantable cardioverter‐defibrillator was implanted in all symptomatic patients. All five asymptomatic patients remained well after they were managed conservatively by strict antipyresis during fever and advice on medication avoidance. ECG screening was performed in all family members and. Two patients had family members with BrS ECG pattern. Genetic test was performed in five patients (62.5%). Two likely pathogenic variants and two variants of unknown significance (VUS) in the SCN5A gene were found in four patients.

**TABLE 1:** Summary of our case series of pediatrics BrS patient.

Patient	Gender	Age at diagnosis (years)	Symptoms/presentation	Fever on presentation	ECG at presentation	Baseline ECG	Coexisting arrhythmia	Flecainide challenge test	Echocardiogram	Holter study	Treadmill test	Treatment	ICD	Family history and ECG screening	Genetic test	Outcome
1	F	4	Aborted cardiac arrest	Yes	Ventricular fibrillation	Multiple chaotic atrial ectopics and frequent PVCs	Ectopic atrial tachycardia	No	Normal	Frequent atrial ectopy, non‐sustained runs of AT, runs of NSVT, infrequent PVCs	Exercise induced non‐sustained monomorphic VT, mainly in recovery phase of exercise	Flecainide, nadolol, transcatheter ablation of atrial tachycardia	Yes	Unremarkable	WES: SCN5A:c.1673A>G (p.H558R) (VUS)	Appropriate shock × 1; inappropriate shock × 4; no recurrence after transcatheter ablation
								Develop type 1 BrS pattern after taking Flecainide								
2	F	0.25	Fever, dyspnea	Yes	Ventricular tachycardia	Prolonged QTc 510 ms	Long QT syndrome	Yes, positive	Repaired perimembranous VSD and ASD—no residual lesion	Normal	Rate dependent RBBB aberrancy, QT variability, normalize recovery phase	Propranolol quinidine	Yes	Father ECG type 1 BrS pattern	NGS: Arrhythmia panel: SCN5A:c.4885C>T (likely pathogenic)	Four episodes of breakthrough VT No recurrence after start of quinidine
3	M	16	Aborted cardiac arrest	No	N/A	Type 1 BrS pattern	N/A	No	Normal	Normal	No exercise induced arrhythmia	No antiarrhythmics	Yes	Parents ECG normal	NGS: BrS panel: SCN5A:c.298_307del (likely pathogenic)	Asymptomatic
4	M	3	Fever and tachycardia	Yes	Type 1 BrS pattern	Incomplete RBBB, no BrS pattern	N/A	No	Normal	Normal	No exercise induced arrhythmia	Conservative management	No	Parents ECG normal	N/A	Asymptomatic
5	M	14	Post COVID vaccination fever	Yes	Type 1 BrS pattern	Type 2 BrS pattern	N/A	No	Normal	Normal	No exercise induced arrhythmia	Conservative management	No	Parents ECG normal	NGS Brugada syndrome panel: Negative	Asymptomatic
6	M	16	Fever and tachycardia	Yes	Type 2 BrS pattern	Type 2 BrS pattern	N/A	Yes, positive	Normal	Normal	N/A	Conservative management	No	Mother ECG—type 2 BrS pattern	NGS: BrS panel: SCN5A:c.4573G>A (VUS)	Asymptomatic
7	M	15	Fever and tachycardia	Yes	Type 1 BrS pattern	Incomplete RBBB, no BrS pattern	N/A	No	Normal	N/A	N/A	Conservative management	No	Unremarkable	N/A	Asymptomatic
8	M	13	Fever and tachycardia	Yes	Type 2 BrS	Type 2 BrS pattern	N/A	Yes, positive	N/A	N/A	No exercise induced arrhythmia	Conservative management	No	Unremarkable	N/A	Asymptomatic

Abbreviations: ASD = atrial septal defect, AT = atrial tachycardia, BrS = Brugada syndrome, ECG = electrocardiogram, F = female, ICD = implantable cardioverter‐defibrillator, M = male, MAT = multifocal atrial tachycardia, N/A = not application, NGS = next generation sequencing, NSVT = non‐sustained ventricular tachycardia, PVCs = premature ventricular contractions, RBBB = right bundle branch block, RVQT = right ventricular outflow tract, VT = ventricular tachycardia, VSD = ventricular septal defect, VUS = variants of unknown significance, WES = whole exome sequencing.

We presented two young female patients with symptomatic BrS, exhibiting additional arrhythmic characteristics. The clinical presentation and dilemma in medical management are discussed in details as follow.

### 3.1 Patient 1

**FIGURE 1:**
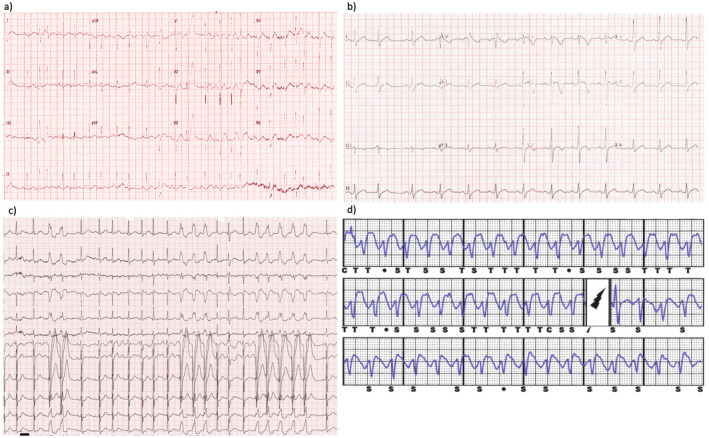
Electrocardiograms (ECGs) and implantable cardioverter‐defibrillator (ICD) electrograms of patient 1. (a) Multiple atrial ectopics and PVCs at presentation. (b) Spontaneous type 1 Brugada ECG pattern on flecainide therapy. (c) Non‐sustained runs of VT. (d) Inappropriate shock due to tachycardia and ST segment changes during febrile illness, with T‐wave oversensing. PVCs, premature ventricular contractions; VT, ventricular tachycardia.

A 4‐year‐old girl survived out‐of‐hospital arrest with documented ventricular fibrillation (VF). She had fever and her ECG showed multifocal atrial ectopics and frequent multifocal premature ventricular contractions (PVCs), with suspected origins from right ventricular outflow tract (RVOT) (Figure 1a). Echocardiography and cardiac magnetic resonance imaging showed normal heart structure and function. Parents refused adrenaline and flecainide challenge test. Subsequently, flecainide and verapamil were started and successfully suppressed the atrial and ventricular arrhythmia. Nevertheless, after initiating treatment with flecainide, the patient developed a type 1 BrS ECG pattern (Figure 1b). Consequently, the medication was switched from flecainide to quinidine. Subcutaneous implantable cardioverter defibrillator (SICD) was implanted for secondary prevention (Kwok et al. 2018). However, quinidine failed to control her atrial and ventricular arrhythmia. She experienced an episode of appropriate ICD shock with ECG demonstrated frequent non‐sustained runs of monomorphic ventricular tachycardia (VT) (Figure 1c). Flecainide was restarted with improved arrhythmia control despite the recurrence of BrS pattern on ECG. A VUS SCN5A:c.1673A>G (p.H558R) was found. She remained asymptomatic for 5 years but her atrial tachyarrhythmia had worsened with time.

At age of nine, she received multiple episodes of inappropriate ICD shock during febrile episodes, due to T wave oversensing because of ST segment elevation (Figure 1d). Flecainide was discontinued but unfortunately patient's atrial arrhythmia worsened. Electrophysiological study was conducted, showing an ectopic atrial tachycardia originating from the lateral mitral annulus. The tachycardia cycle length was measured to be between 200 and 300 ms. Radiofrequency ablation of ectopic atrial focus was successful. PVCs and VT were suppressed after general anesthesia. Endocardial pace mapping failed to localize the possible exit site, suggesting epicardial origin of ventricular substrate. She had no syncope or palpitation after the ablation, and follow up treadmill test showed no exercise induced arrhythmia.

### 3.2 Patient 2

**FIGURE 2:**
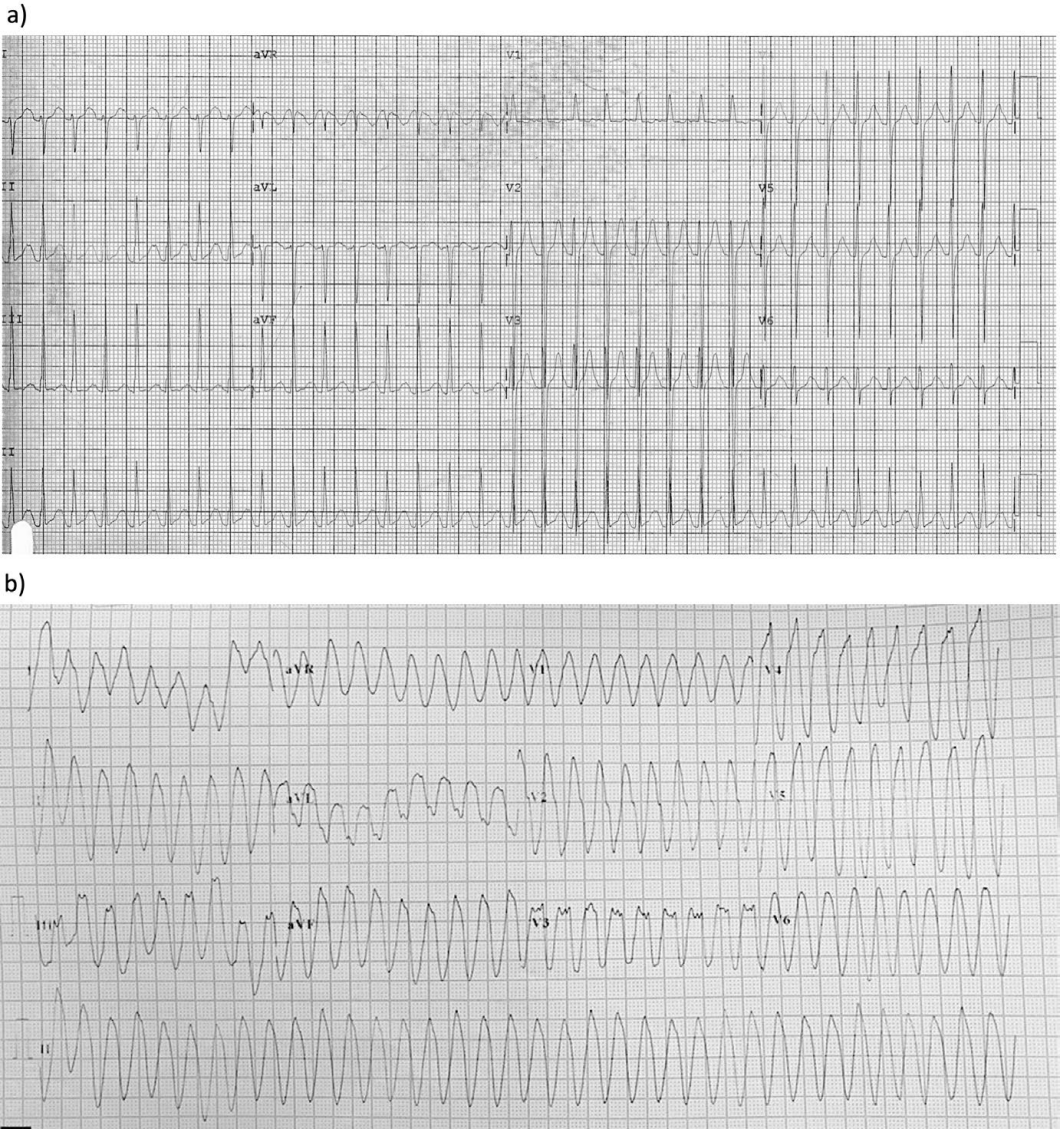
Electrocardiograms (ECGs) of patient 2. (a) Baseline ECG showing prolonged QTc and (b) breakthrough monomorphic ventricular tachycardia (VT).

A 3‐month‐old girl with a history of repaired perimembranous ventricular septal defect (VSD) and atrial septal defect (ASD) presented with fever and shortness of breath. ECG showed polymorphic VT requiring cardioversion. Pre‐operative ECG before VSD and ASD repair was reviewed which showed prolonged QTc up to 510 ms (Figure 2a). There was no BrS ECG pattern. She was started on propranolol for suspected long QT syndrome (LQTS).

She experienced two episodes of monomorphic VTs during intercurrent febrile illness at 17 months and 4 years of age (Figure 2b). Epicardial ICD was implanted. Subsequent flecainide challenge test demonstrated marked bradycardia, wide QRS complex and type 1 BrS ECG pattern. Her father's ECGs also showed type I BrS pattern. Genetic testing revealed a likely pathogenic variant in SCN5A gene, c.4885C>T (p.Arg1629*). She was started on quinidine with close monitoring of the QT interval. With the combination of quinidine and propranolol, she has been event‐free for 5 years. Follow‐up ECGs showed a QTc interval of 450 ms.

## 4 Discussion

### 4.1 Demographics of Pediatric BrS

Over the past 20 years, our center had eight children with BrS. This low prevalence is not surprising, as BrS typically manifests in individuals in their fourth decade of life (Coppola et al. 2021). A multicenter study by Probst et al. (2007) spanning 15 years and involving 13 different institutions identified only 30 pediatric BrS patients. A large‐scale Japanese study screened asymptomatic children and found a prevalence of Brugada‐type ECG of only 0.0098%, in stark contrast to the reported prevalence of BrS in adults, which can be as high as 1 in 2000 (Coppola et al. 2021; Yamakawa et al. 2004) The disparity in prevalence is believed to be linked to the low level of testosterone in children (Yamakawa et al. 2004). The potential role of testosterone in relation to BrS ECG pattern was first highlighted when two adult patients experienced the disappearance of their Brugada ECG following castration (Matsuo et al. 2003). Later the pediatric study by Yamakawa et al. (2004) demonstrated that the prevalence of Brugada‐like ECG patterns in children increases with the onset of puberty. This study explained the absence of a male predominance in pediatric cohorts, if the low testosterone level in children play a significant role (Probst et al. 2007; Gonzalez Corcia et al. 2017). In our study, there were more male patients and five out of six male patients were in the post‐pubertal age and suggesting the influence of testosterone in BrS.

### 4.2 Fever and Pediatric BrS

Both children and adult studies have identified a history of syncope, spontaneous BrS type 1 ECG pattern, and the presence of atrial arrhythmias as predictors of arrhythmic events (Coppola et al. 2021; Gonzalez Corcia et al. 2017). However, the prognosis of fever‐induced BrS in asymptomatic children remains uncertain. According to the findings of a study conducted by Mizusawa et al. (2016) in adults, patients with fever‐induced BrS type 1 ECG pattern exhibited an arrhythmic event rate of 0.9% per year. This rate is comparable to that of initially asymptomatic patients with a spontaneous BrS type 1 ECG pattern, which ranges from 0.5% to 0.8% per year. In a pediatric study conducted by Gonzalez Corcia et al. (2017), individuals who presented with a fever‐induced BrS ECG pattern were categorized as having a spontaneous pattern, which was linked to a more unfavorable prognosis. However, recent research has indicated that the fever‐induced BrS ECG pattern may be less specific than previously thought, as the type 1 pattern was found to be 20 times more prevalent in febrile patients compared to afebrile patients (Adler et al. 2013). It is possible that the long‐term risk of fever‐induced type 1 BrS ECG pattern in asymptomatic children may be overestimated, as it could potentially be an incidental finding. In our cohort, all five asymptomatic patients who displayed type 1 BrS ECG pattern during febrile illness had a favorable prognosis with conservative management involving strict antipyresis and avoidance of triggering medications. Further studies are necessary to evaluate the risk of arrhythmic events in asymptomatic children with fever‐induced type 1 BrS ECG pattern. This is essential to avoid overtreatment and unnecessary emotion burden on patients and their families.

### 4.3 Coexisting Arrhythmia of Pediatric BrS

Some BrS patients may present with multiple arrhythmias simultaneously, therefore some studies described these as “overlapping syndrome.” (Coppola et al. 2021) Various arrhythmias have been reported to coexist with BrS, including atrial fibrillation, sinus node dysfunction, long QT syndrome, and short QT syndrome (Hayashi et al. 2018; Bezzina et al. 1999). In our study, two patients were diagnosed with BrS and coexisting arrhythmias.

Atrial arrhythmias, particularly atrial fibrillation and atrial flutter, have been observed in up to 38% of adult patients with BrS (Probst et al. 2007; Pierre et al. 2004). The prevalence of coexisting atrial arrhythmias in childhood BrS is relatively low, with reported rates of 8%–10% (Probst et al. 2007; Gonzalez Corcia et al. 2017). Underestimation of atrial arrhythmias in pediatric patients with BrS is possible, as these patients may be asymptomatic, similar to findings in adult studies (Probst et al. 2007). Coexisting atrial arrhythmias in adults with BrS may indicate a higher risk of sudden death (Pierre et al. 2004). Research has shown that atrial arrhythmia is a significant clinical variable predicting lethal events in pediatric patients (Gonzalez Corcia et al. 2017). Therefore, it is important for clinicians to consider providing Holter surveillance as part of risk stratification in pediatric patients with BrS, in order to detect atrial arrhythmias.

Long QT syndrome (LQTS) can coexist with BrS (Coppola et al. 2021). Flecainide has been found to shorten the QT interval in patients with SCN5A variants (Benhorin et al. 2020). However, the use of flecainide in these patients raises safety concerns as it can unmask the underlying BrS and predispose patients to have ventricular arrhythmia. On the other hand, quinidine has been used in adult BrS patients to suppress ventricular arrhythmias and hence, it has been suggested to be use as a “bridge to ICD” (Belhassen, Glick, and Viskin 2004) However, its safety in children has not been extensively assessed. This is particularly important to consider due to the potential coexistence of LQTS, which can be exacerbated by quinidine and potentially lead to the development of torsades de pointes (Baruteau, Mabo, and Probst 2009). Our BrS patient with coexisting LQTS who was on quinidine showed its efficacy in preventing potentially lethal events with the QT interval closely monitored.

The presence of coexisting arrhythmias presents challenges in determining the most appropriate medical treatment, as demonstrated by the two cases discussed. In some instances, anti‐arrhythmic medications that initially appears contraindicated might ultimately be necessary to manage the underlying arrhythmia. The selection of medication should be specific to the observed malignant arrhythmia, while closely monitoring for potential adverse effects, such as QT interval prolongation or ventricular tachyarrhythmias.

## 5 Conclusion

Fever is a major factor unmasking BrS in children. Asymptomatic children who exhibited only type 1 BrS ECG pattern during episodes of fever demonstrated a favorable prognosis when managed conservatively. Children with Brugada syndrome and coexisting arrhythmias may face increased difficulty in achieving effective control of their arrhythmias.

## Author Contributions

Hei‐To Leung and Sit‐Yee Kwok conceived of the presented idea. Hei‐To Leung and Sit‐Yee Kwok wrote the manuscript with the support from Kin‐Shing Lun, Tak‐Cheung Yung and Sabrina Tsao. Fong‐Ying Shih contributed to sample preparation. Hei‐To Leung analyzed the data. All authors discussed the results and contributed to the final manuscript.

## Conflicts of Interest

The authors declare no conflicts of interest.

## Statement Relating to Ethics and Integrity Policies

The authors agreed with the ethics and integrity policies.

## Data Availability

The data from the findings of this study were inferred can be obtained from the corresponding author upon reasonable request. All data and materials support the published claims and comply with field standards.
